# A New Procedure in Dystocia

**Published:** 1891-06

**Authors:** J. W. Thomson

**Affiliations:** New York


### A NEW PROCEDURE IN DYSTOCIA.

J. W. Thomson, M. D., New York.

On December 30th, 1890, called in consultation by Dr. M. in a case of dystocia. Dr. F. had also been called and was in attendance. The child lay in the second position of descent of the right shoulder, and had been dead from eight to nine hours. The liquor amnii had broken away at about ten o'clock on the previous morning. It was from three to four hours afterward before the physician in attendance had been called. They had tried to get along with an *experienced nurse.* The indicated remedy had been exhibited, and repeated efforts made to bring down the feet; all attempts, however, at version proved abortive. We each essayed it after my arrival, but the child was so thoroughly wedged that it was found impossible to get a hand up sufficiently to grasp the feet. In all probability it was too late for the successful performance of this operation when Dr. M. was called. Chloroform had been and was being administered, but all to no purpose. The uterus would not relax. Even had we been able to grasp the feet of the dead babe, the uterus was so tensely contracted that I feared rupture. I protested against the administration of Chloroform. The reply was that she must have something to relieve her sufferings. She was praying for death. The agony could not be much longer borne. To take the child away piece-meal was thought to be the only alternative. It was feared that the mother could not endure the shock and the waiting much longer.

I resolved to attempt what would have been impossible; or, at least what I would not have thought of attempting had the child been alive. I ordered that no more Chloroform be administered. I desired the woman perfectly conscious that her vital force might fully respond. My thought was to bring the head from the right iliac fossa to the pubis. In the interval I introduced my right hand under the body of the child, the left grasping the arm at the shoulder. When the contractions began I pushed carefully yet firmly upward with my right hand, and with the left pulled downward and forward, striving to operate in the direction of the pelvic axis. The uterine contractions, which had hitherto been weak and feeble, became strong and forcible, as though they felt and responded to the stimulus. The mother held her breath. The child moved. When the pains ceased the head was compressed under the right side of the pubic arch. The body of the child had risen in the direction of the superior strait. During the next contractile efforts I supported the perineum with my left hand, striving to compress the head as much as possible with my right hand, and the head was born. There was joy when I announced success. The danger was past. The next pains brought the poor dead babe into the world. There was no rupture. I saw the woman about a month afterward. She made a good recovery.

